# Supplementary material for: The same biophysical mechanism is involved in both temporal interference and direct kHz stimulation of peripheral nerves
Source: Nat Commun. 2025 Oct 9;16:9006. doi: 10.1038/s41467-025-64059-w (PMC12511453; doi:10.1038/s41467-025-64059-w)
Supplement: Supplementary file 1 — Supplementary Information [file 41467_2025_64059_MOESM1_ESM.pdf]

Supplementary information

The same biophysical mechanism is involved in both temporal interference and direct kHz stimulation of peripheral nerves

Aleksandar Opančar,[1,2] Petra Ondráčková,[1,3] David Samuel Rose,[1] Jan Trajlinek,[1,4] Vedran Đerek,[2] Eric Daniel Głowacki[1,5*]

[1] Bioelectronics Materials and Devices Lab, Central European Institute of Technology CEITEC, Brno University of Technology, Purkyňova 123, 61200 Brno, Czech Republic

[2] Department of Physics, Faculty of Science, University of Zagreb, Bijenička c. 32, 10000, Zagreb, Croatia

[3] Institute of Scientific Instruments of the CAS, v. v. i., Královopolská 147, 61264 Brno, Czech Republic

[4] International Clinical Research Center, St. Anne's University Hospital Brno, Brno 60200, Czech Republic

[5] Department of Biomedical Engineering, Faculty of Electrical Engineering and Communication, Brno University of Technology, Technická 12, 61600 Brno, Czech Republic

Corresponding email: E-mail: glowacki@vutbr.cz

Supplementary Note 1
Thresholds comparison between 2 and 4-electrode configurations

Directly comparing 4-electrode TIS threshold to the thresholds obtained with two electrode configurations is generally not possible since the different configurations will not produce the same stimulus amplitude (activating function) at the nerve location for the same stimulation current. We have measured the 4-electrode TIS to make sure we observe the same general trend in the S-F relationship, but the direct threshold comparisons should only be made considering 2-electrode configurations, that is between the unmodulated and modulated sine since we measure them in same configurations and they can thus be directly compared.

The effect of the configuration on the current threshold is a complicated issue which needs to be modeled with proper finite element techniques and realistic tissue and contact impedances. We will try to demonstrate the concept, however, using a simplified model that can be solved analytically to try to build intuition and to get a sense of the important parameters.

For this purpose, we will assume a semi-infinite tissue of uniform electrical conductivity σ with point current sources for electrodes. We will compare the two configurations and see in which cases each configuration produces the larger stimulus at the target location. The two configurations in this simplified hemispheric geometry are depicted in the figure below with the nerve depicted in orange propagating perpendicular to the depicted plane:

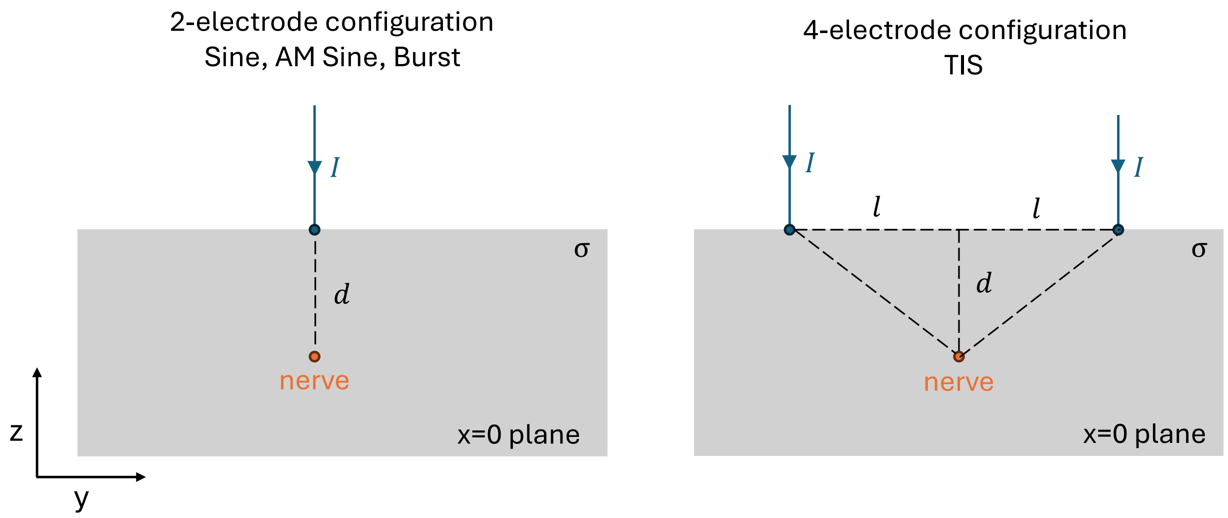

Since the tissue is isotropic for the bottom hemisphere, the symmetry results in isotropic current density of magnitude:

$$J(r) = \frac{I}{2\pi r^2}$$

Where r is the distance from the current source. Ohms law $J = \sigma E$ allows us to find the expression for the electric field and electric potential.

$$E = \frac{J}{\sigma} = \frac{I}{2\pi\sigma r^2} = -\frac{\mathrm{d}V(r)}{\mathrm{d}r}$$

$$V = \frac{I}{2\pi\sigma r}$$

Finally, we can find the expression for activating function in the nerve direction (labeled x direction for consistency with the manuscript) evaluated on the depicted plane:

$$A_x = \left.\frac{d^2V}{dx^2}\right|_{x=0} = -\frac{I}{2\pi\sigma r^3}$$

If we want to have the same activating functions at the nerve location in both configurations for the same stimulation current I, we must have:

$$\frac{I}{2\pi\sigma d^3} = \frac{I}{2\pi\sigma(l^2 + d^2)^{3/2}} + \frac{I}{2\pi\sigma(l^2 + d^2)^{3/2}}$$

Which gives the solution:

$$l = \sqrt{2^{2/3} - 1}\, d \approx 0.77\, d$$

Only in the special geometry where this condition is satisfied will both configurations give the same activating function at the nerve location. If the electrodes are farther apart (or equivalently the nerve shallower), the additional distance from the nerve in the 4-electrode configuration will not be fully compensated by 2x the injected current (one I through each electrode pair) and the 2-electrode configuration will have apparently lower stimulation threshold. If the electrodes are closer (or the nerve deeper) the 4-electrode configuration will have the advantage and it will have lower threshold.

Supplementary Note 2

Derivation for the TOT expression (1):

For sinusoidal stimulation it is possible to transform the data from frequency domain to time domain. The electric potential and the activating function in x direction $A = d^2V/dx^2$ are both phasor quantities that are fully characterized by their amplitude and phase (and frequency which is known). The amplitudes A_1, A_2 and phases φ_1, φ_2 are time independent, but it is possible to write down the expressions for time dependent activation functions:

$$A_1(t) = A_1 \cdot \cos\,(\omega_1 t + \varphi_1)$$

$$A_2(t) = A_2 \cdot \cos\,(\omega_2 t + \varphi_2)$$

That gives the total time dependent activation function as:

$$A(t) = A_1(t) + A_2(t) = A_1 \cdot \cos(\omega_1 t + \varphi_1) + A_2 \cdot \cos\,(\omega_2 t + \varphi_2)$$

For the purposes of calculating TOT, we are interested in the envelope of the resulting activating function $A(t)$ which we will term $E(t)$. We can find the expression for $E(t)$ from simple geometric considerations by representing the quantities $A_1(t)$ and $A_2(t)$ as rotating vectors in the complex plane:

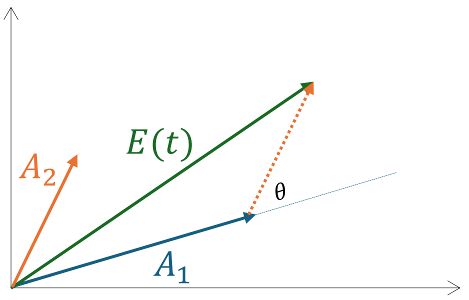

We can use the law of cosines to obtain the time dependent envelope $E(t)$ while using the identity $\cos(180 - \theta) = -\cos\theta$

$$E(t) = \sqrt{A_1^2 + A_2^2 + 2A_1A_2\cos\theta}$$

The angle θ will equal the phase difference between A_2 and A_1:

$$\theta = \omega_2 t + \varphi_2 - (\omega_1 t + \varphi_1) = (\omega_2 - \omega_1)t + \Delta\varphi$$

Where $\Delta\phi \equiv \varphi_2 - \varphi_1$

The time dependent expression for E(t) then becomes:

$$E(t) = \sqrt{A_1^2 + A_2^2 + 2A_1A_2\cos((\omega_2 - \omega_1)t + \Delta\varphi)}$$

Time over threshold can then be found by calculating the time difference between two consecutive solutions of the equation $E(t) = A_T$:

$$\sqrt{A_1^2 + A_2^2 + 2A_1A_2\cos((\omega_2 - \omega_1)t + \Delta\varphi)} = A_T$$

Which after squaring both sides and rearranging gives:

$$\cos((\omega_2 - \omega_1)t + \Delta\varphi) = \frac{A_T^2 - A_1^2 - A_2^2}{2A_1A_2}$$

Solving this equation gives:

$$(\omega_2 - \omega_1)t + \Delta\varphi = \pm\arccos\left(\frac{A_T^2 - A_1^2 - A_2^2}{2A_1A_2}\right) + 2k\pi \qquad k \in \mathbb{Z}$$

And the difference between the two consecutive solutions for t is then:

$$\Delta t = \frac{2}{\omega_2 - \omega_1}\arccos\left(\frac{A_T^2 - A_1^2 - A_2^2}{2A_1A_2}\right) \qquad \text{if} \qquad |A_1 - A_2| \le A_T \le A_1 + A_2$$

The definition for the TOT that we give in the manuscript is “the fraction of each modulation period that the nerve is suprathreshold” so to obtain the expression for TOT we need to divide Δt by the modulation period which is $T_{MOD} = \frac{2\pi}{\omega_2 - \omega_1}$ which gives the solution given in expression (1).
