# Supplementary material for: The same biophysical mechanism is involved in both temporal interference and direct kHz stimulation of peripheral nerves
Source: Nat Commun. 2025 Oct 9;16:9006. doi: 10.1038/s41467-025-64059-w (PMC12511453; doi:10.1038/s41467-025-64059-w)
Supplement: Supplementary file 2 — Description of Additional Supplementary Files [file 41467_2025_64059_MOESM2_ESM.pdf]

Description of Additional Supplementary Files

Supplementary Movie 1: Example video depicting how only subtle movement of electrode positioning over the target area can cause a change from phasic to tonic stimulation. Before electrode movement the AM hotspot is positioned over the target area, when one of the electrodes is moved directly over the target area, the area is now exposed largely to unmodulated kHz signals and thus is tonically stimulated. No stimulation parameters, except electrode positioning, were changed.
