# Supplementary material for: The same biophysical mechanism is involved in both temporal interference and direct kHz stimulation of peripheral nerves
Source: Nat Commun. 2025 Oct 9;16:9006. doi: 10.1038/s41467-025-64059-w (PMC12511453; doi:10.1038/s41467-025-64059-w)
Supplement: Supplementary file 4 — Reporting Summary [file 41467_2025_64059_MOESM4_ESM.pdf]

nature portfolio

Corresponding author(s): Eric Daniel Glowacki

Last updated by author(s): Aug 6, 2025

Reporting Summary

Nature Portfolio wishes to improve the reproducibility of the work that we publish. This form provides structure for consistency and transparency in reporting. For further information on Nature Portfolio policies, see our Editorial Policies and the Editorial Policy Checklist.

Statistics

For all statistical analyses, confirm that the following items are present in the figure legend, table legend, main text, or Methods section.

n/a	Confirmed	
☐	☒	The exact sample size (*n*) for each experimental group/condition, given as a discrete number and unit of measurement
☐	☒	A statement on whether measurements were taken from distinct samples or whether the same sample was measured repeatedly
☐	☒	The statistical test(s) used AND whether they are one- or two-sided<br>*Only common tests should be described solely by name; describe more complex techniques in the Methods section.*
☐	☒	A description of all covariates tested
☐	☒	A description of any assumptions or corrections, such as tests of normality and adjustment for multiple comparisons
☐	☒	A full description of the statistical parameters including central tendency (e.g. means) or other basic estimates (e.g. regression coefficient) AND variation (e.g. standard deviation) or associated estimates of uncertainty (e.g. confidence intervals)
☐	☒	For null hypothesis testing, the test statistic (e.g. *F*, *t*, *r*) with confidence intervals, effect sizes, degrees of freedom and *P* value noted<br>*Give P values as exact values whenever suitable.*
☒	☐	For Bayesian analysis, information on the choice of priors and Markov chain Monte Carlo settings
☒	☐	For hierarchical and complex designs, identification of the appropriate level for tests and full reporting of outcomes
☒	☐	Estimates of effect sizes (e.g. Cohen's *d*, Pearson's *r*), indicating how they were calculated

Our web collection on statistics for biologists contains articles on many of the points above.

Software and code

Policy information about availability of computer code

Data collection	PicoScope 7 v7.1.39.3737, PsychoPy v2024.2.4, Python 3.13, Tracker v6.3.0,<br>COMSOL Multiphysics 6.3
Data analysis	OriginPro 2024 (64-bit) SR1 10.1.0.178; Python 3.13

For manuscripts utilizing custom algorithms or software that are central to the research but not yet described in published literature, software must be made available to editors and reviewers. We strongly encourage code deposition in a community repository (e.g. GitHub). See the Nature Portfolio guidelines for submitting code & software for further information.

Data

Policy information about availability of data

All manuscripts must include a data availability statement. This statement should provide the following information, where applicable:

- Accession codes, unique identifiers, or web links for publicly available datasets
- A description of any restrictions on data availability
- For clinical datasets or third party data, please ensure that the statement adheres to our policy

All data supporting the findings of this study are available within the article, and both raw and processed data source data is available at https://doi.org/10.6084/m9.figshare.28535426. Any additional requests for information can be directed to, and will be fulfilled by, the corresponding author.

Research involving human participants, their data, or biological material

Policy information about studies with human participants or human data. See also policy information about sex, gender (identity/presentation), and sexual orientation and race, ethnicity and racism.

Reporting on sex and gender	For the human volunteer groups, the sex and age were recorded, and these details are reported in the manuscript methods section. Sex was not considered as a variable in the locust experiments.
Reporting on race, ethnicity, or other socially relevant groupings	N/A
Population characteristics	Subjects were recruited by advertisement on the university campus, and were healthy volunteers who submitted written consent. Participants were recruited via an advertisement in the local community and on the institute's campus. A total of N=8 participants (average age 34.25 ± 11.2, 62% Female) were recruited for the motor stimulation of the median nerve. For our afferent nerve stimulation experiments N=11 participants (average age = 36.3 ± 8.21, 36% Female) were recruited. For the determination of the 'U-curve' in the afferent model N=6 participants were recruited (average age = 33 ± 10, 83% Female); for the efferent model N=7 participants (average age = 27 ± 5, 28% female)
Recruitment	Recruitment of volunteers was sollicited at the CEITEC VUT university campus, as described in the methods section. Therefore the subject pool was predominated by students, thus a generally younger population segment. For the purpose of the nerve excitability experiments in this paper, we do not believe the variable of age or other factors to be meaningful.
Ethics oversight	Eticka komise CEITEC VUT pro vyzkum za ucasti lidskych subjektu (Ethics Committee of CEITEC Brno University of Technology for research on volunteer human subjects) approved the protocol.

Note that full information on the approval of the study protocol must also be provided in the manuscript.

Field-specific reporting

Please select the one below that is the best fit for your research. If you are not sure, read the appropriate sections before making your selection.

☒ Life sciences ☐ Behavioural & social sciences ☐ Ecological, evolutionary & environmental sciences

For a reference copy of the document with all sections, see nature.com/documents/nr-reporting-summary-flat.pdf

Life sciences study design

All studies must disclose on these points even when the disclosure is negative.

Sample size	The sample sizes of locusts, or humans, was N=5-ll, depending on the given experiment. The variables being tested were current thresholds for nerve excitability, and the intersubject variability affecting these experiments is low, as can be appreciated by the standard error of the means reported throughout the experiments. No other special measures were taken to calculate samples sizes, as the variable being tested as not a biological one, but a physical one. We would note that sample sizes for nerve excitability studies where electrical stimulation parameters are the variable under test have similar sample sizes of up to 10 individuals.
Data exclusions	No data exclusion was performed for any reason. At the level of analyzing data, when assessing the significance in current threshold, differences between 4- and 2-electrode stimulation arrangements, 4-electrode data were not considered together with the 2-electrode data. We describe in the manuscript why this would be misleading, as the electrode spatial configuration is very different, and the total magnitude of injected current is always higher in 4-electrode configuration. This is covered in detail in Appendix 1.
Replication	All experiments done for the revised version of this manuscript were replicated accross N=5-11, depending on the experiment. The number of participants in the first submitted version was lower, N=3. Nevertheless the conclusions from both the first study and the refined second study are the same, which strengthens the case for reproducibility of our findings, i.e. the experimental findings were replicated.
Randomization	Presentation of carrier frequencies and amplitude modulation frequencies was randomized by software in all trials, locust and human.
Blinding	Both experimenter and subject were blinded as to the signal frequency/amplitude being presented - this is software controlled.

Reporting for specific materials, systems and methods

We require information from authors about some types of materials, experimental systems and methods used in many studies. Here, indicate whether each material, system or method listed is relevant to your study. If you are not sure if a list item applies to your research, read the appropriate section before selecting a response.

Materials & experimental systems

n/a	Involved in the study
☒	☐ Antibodies
☒	☐ Eukaryotic cell lines
☒	☐ Palaeontology and archaeology
☐	☒ Animals and other organisms
☒	☐ Clinical data
☒	☐ Dual use research of concern
☒	☐ Plants

Methods

n/a	Involved in the study
☒	☐ ChIP-seq
☒	☐ Flow cytometry
☒	☐ MRI-based neuroimaging

Animals and other research organisms

Policy information about studies involving animals; ARRIVE guidelines recommended for reporting animal research, and Sex and Gender in Research

Laboratory animals	Locusta migratoria
Wild animals	N/A
Reporting on sex	Sex was not considered as a variable, both male and female locusts were used and the sex was not recorded.
Field-collected samples	N/A
Ethics oversight	Experiments on locusts, as invertebrates, do not fall under legislation in the Czech Republic as animal experiments, therefore no special ethical permission is required (Act No. 246/1992 Sb.)

Note that full information on the approval of the study protocol must also be provided in the manuscript.

Plants

Seed stocks	N/A
Novel plant genotypes	N/A
Authentication	N/A
